# The relationship between serum ghrelin levels and hair zinc concentrations in children

**DOI:** 10.4008/jcrpe.v1i1.14

**Published:** 2010-12-08

**Authors:** Selim Kurtoğlu, Esad Köklü, Nihal Hatipoğlu, Mehmet Emre Atabek

**Affiliations:** 1 Erciyes University, Faculty of Medicine, Department of Pediatrics, Division of Pediatric Endocrinology & Metabolism, Kayseri, Turkey; +90-352 438 00 76+90-352 437 58 25selimk@erciyes.edu.trErciyes Üniversitesi, Tıp Fakültesi, Çocuk Sağlığı ve Hastalıkları AD, Neonatoloji ve Pediatrik Endokrinoloji ve Metabolizma Bölümü-Kayseri-Turkey

**Keywords:** growth, Zinc, ghrelin

## Abstract

**Background:** Zinc (Zn) plays a central role in the activation of numerous enzyme systems that synthesize and degrade bioactive peptides. Some of these bioactive peptides, also called neuropeptides, are involved in the regulation of food intake.

**Objective:** In this study we aimed to demonstrate the relationship between serum ghrelin and hair Zn concentrations in children.

**Methods:** Prepubertal children brought to our outpatient clinics by their parents because of signs and symptoms of pica, poor appetite, poor growth, and other complaints were included in the study. The children were divided into two groups according to Zn hair concentrations. Group 1 consisted of children with low (< 70 μg/g) hair Zn levels, and group 2 of children with normal ( ≥ 70 μg/g) hair Zn levels. Hair Zn concentrations, serum ghrelin, insulin-like growth factor I (IGF-I) and IGF-binding protein-3 (IGFBP-3) levels were measured in all children.

**Results:** There were 10 children with low hair Zn levels (group 1) and 15 with normal levels (group 2). Serum IGF-I, IGFBP-3 and ghrelin concentrations of group 1 (103.1 ± 71.8 ng/mL, 1412.8 ± 615.7 ng/mL and 0.96 ± 0.22 ng/mL, respectively) were lower than in group 2(164.9 ± 40.5 ng/mL, 2398.5 ± 295.5 ng/mL and 1.21 ± 0.23 ng/mL, respectively). In univariate analysis, Zn hair concentration was positively associated with serum IGF-I (r=0.424, p=0.035) and IGFBP-3 (r=0.671, p < 0.001) concentrations. The correlation between ghrelin and hair Zn concentrations was not significant (r=0.202, p=0.333).

**Conclusion:** Serum ghrelin concentrations might be affected by low hair Zn concentrations in children.

**Conflict of interest:**None declared.

## INTRODUCTION

Ghrelin, a 28 aminoacid peptide that is the natural endogenous ligand for growth hormone (GH) secretagogue receptors (GHS-Rs), was originally isolated from rat and human stomach(1) and was subsequently identified in various tissues, including small bowel, pancreas, kidney, pituitary, and hypothalamus.(2, 3) The finding that GHS-Rs are present in several brain areas and in peripheral tissues suggests a regulatory role for this brain–gut peptide in many endocrine and nonendocrine biological activities.(4) Indeed, in addition to acting as a potent GH releaser, ghrelin stimulates both prolactin and adrenocorticotropic hormone secretion, shows an orexigenic effect, and plays a role in energy homeostasis.(4) Caloric intake and chronically positive energy balance suppress ghrelin secretion and mRNA expression, whereas weight loss and restriction of caloric intake increase ghrelin expression and secretion.(5, 6) In humans, ghrelin circulating levels are decreased in obesity and, conversely, are elevated in anorexia nervosa and in cachexia due to chronic heart failure (4, 6, 7). However, subnormal ghrelin levels have recently been found in a series of patients with malnutrition due to short bowel syndrome, and this decrease has been ascribed to the reduction in the tissue mass that is able to secrete ghrelin.(8)

Zinc (Zn) deficiency was initially discovered in humans and reported by Prasad et al.(9). Symptoms reported to accompany Zn deficiency included dwarfism, hypogonadism and poor appetite. In these studies, similarities were noted between Zn-deficient human subjects and known characteristics of Zn-deficient animals. The young growing rat is very responsive to the consumption of a Zn-deficient diet. Within 3-5 days, food intake is first observed to decrease. This decrease in appetite is the first visible sign of Zn deficiency and occurs well in advance of any other symptoms associated with Zn deficiency. The reduction in growth associated with Zn deficiency is largely caused by the reduction in intake due to this decrease in appetite.(10) The appetite regulation system consists of both peripheral and central systems. Feedback from the periphery to the brain involves neural feedback through the vagus nerve as well as feedback from blood-borne factors, including both metabolites and hormones.(10) Zinc plays a central role in the activation of numerous enzyme systems that synthesize and degrade bioactive peptides. Some of these bioactive peptides, also called neuropeptides, are involved in the regulation of food intake, and a few possible candidates such as neuropeptide Y, cholecystokinin, melanin-concentrating hormone, ghrelin, and serotonin have been suggested.(11) To our knowledge there are no in vitro or animal studies suggesting involvement of Zn in ghrelin synthesis or secretion in the literature published in English. We, therefore, aimed to investigate the relationship between serum ghrelin levels and hair Zn concentrations in children.

## MATERIALS AND METHODS

The study was carried out in the Paediatric Endocrinology and Metabolism Department of Gevher Nesibe Hospital between January 2003 and 2004. Prepubertal children brought to the outpatient clinics by their parents because of signs and symptoms of pica, poor appetite, poor growth, and other complaints were included in the study. Children with known endocrinological and other chronic diseases such as coeliac disease, liver and renal failure were excluded. Standing height (cm) was measured on a portable stadiometer, calibrated with a machined meter rod. Weight (kg) was determined on electronic scales. Height and weight standard deviation scores (SDS) were calculated from the 1978 age- and sex- specific normal anthropometric data.(12)

Zn status was determined by Zn in hair. Hair samples, 3-4 cm in length, were cut close to the scalp in the occipital region of the head and processed by a modification of the method of McBean, et al.(13) Hair was washed in individual plastic containers with a metal-free 1% detergent by shaking for 10 min, followed by 10 rinses with deionized water. Subsequently, the hair was blotted on filter paper, dried in an oven at 70°C, and stored in a desiccator until weighed for analysis. Approximately 250 mg of dried hair sample was wet ashed in a 4:1 mixture of 65% nitric acid and 70% perchloric acid, and the digest was analyzed by an atomic absorption spectrophometer (Perkin-Elmer Model 403, Perkin-Elmer Corporation, Norwalk, CT) at 213 nm and a slit width of 0.7 nm. Serum IGFBP-3 and IGF-I concentrations were measured by radioimmunoassay (RIA) (Biocode 1014, Biocode 1010, respectively) and the results were expressed in ng/mL. On the day of assessment, the blood sample was drawn between 09:00 and 11:00 AM, at fasting. Serum samples were separated and stored at -70°C. Serum ghrelin concentrations were measured using a commercially available radioimmunoassay (RIA) kit (Phoenix Pharmaceuticals Inc, Phoenix, AZ, USA) that employs 125I-labeled bioactive ghrelin as a tracer and a rabbit polyclonal antibody against full-length octanoylated human ghrelin. The assay detects both ghrelin and desoctanoyl-ghrelin. The sensitivity of the assay is 30 pg/mL. Intra- and inter-assay coefficients of variation are <5% and <14%, respectively.

The children included in the study were divided into two groups according to their hair Zn concentrations. Children with low Zn hair levels (<70 μg/g)(14) were included in group 1; and those with ≥70 μg/g levels, in group 2.

The parents and their children were informed about the aims and design of the study and consent was obtained from the participitants.

## STATISTICAL ANALYSIS

Data are given as mean±SD values. Levels of ghrelin, IGF-I, IGFBP-3 and Zn in hair were tested for normality of distribution by the Kolmogorov-Smirnov test. Differences between groups were evaluated by Student’s independent samples t tests. Chi-squared tests were used to evaluate within-group changes in proportions. Univariate associations between the study variables were analyzed by calculating Pearson’s correlation coefficients. Stepwise linear regression analyses were performed to determine the independent contributions of ghrelin to the variations of IGF-I, IGFBP-3, Zn in hair, age, sex, height and weight SDS. All statistics in this study were done using SPSS 10.0 for Windows.

## RESULTS

Twenty-five children were included in the study. There were 10 children with low Zn in hair (group 1) and 15 children with Zn hair levels of ≥70 μg/g (group 2). The anthropometric data of the groups are summarized in Table 1. Height and weight SDS, age and sex variables were not different between groups 1 and 2 (p>0.05). Serum IGF-I, IGFBP-3 and ghrelin concentrations of group 1 (103.1±71.8 ng/mL, 1412.8±615.7 ng/mL and 0.96±0.22 ng/mL, respectively) were lower as compared to group 2 (164.9±40.5 ng/mL, 2398.5±295.5 ng/mL and 1.21±0.23 ng/mL, respectively), as shown in Table 1. Mean ghrelin concentrations were not different between males (n=13, 1.13±0.25 ng/mL) and females (n=12, 1.08±0.27 ng/mL, p=0.593). Ghrelin concentrations also did not differ between males and females in groups 1 and 2 (data not shown) (p>0.05).

In univariate analyses; hair Zn concentration was positively associated with serum IGF-I (r=0.424, p=0.035) and IGFBP-3 (r=0.671, p<0.001) concentrations, but not with age, sex, height and weight SDS (p>0.05). The association between ghrelin and Zn in hair concentrations was not significant (r=0.202, p=0.333). The correlations between ghrelin and age, sex, height and weight SDS were not significant (p>0.05). Ghrelin concentration was insignificantly associated with serum IGF-I (r=0.347, p=0.089) and IGFBP-3 (r=0.374, p=0.065) concentrations. Stepwise linear regression analysis did not reveal additional significant associations (p>0.05).

**Table 1:**
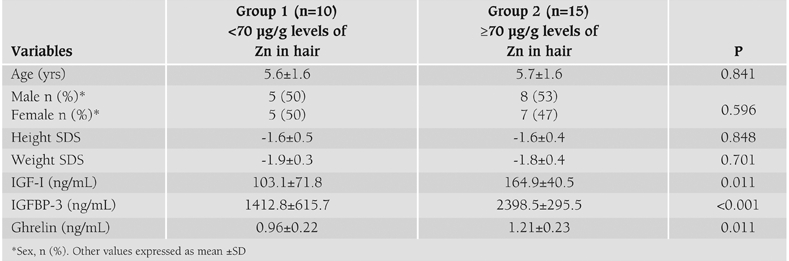
Anthropometric data and serum IGF-I, IGFBP-3 and ghrelin concentrations in the two groups

## DISCUSSION

This study investigated the relationship between serum ghrelin and hair Zn concentrations in children. Although it has been known for more than 50 years that Zn deficiency regularly and consistently causes anorexia in several animal species, the basic mechanism that causes this phenomenon remains an enigma. Zinc plays a central role in the activation of numerous enzyme systems that synthesize and degrade bioactive peptides.(11) The hypothalamus and other brain regions controlling energy homeostasis contain neuronal populations that produce specific neuropeptides that have effects on feeding behaviour and body weight.(15) Zn levels were found to influence gene expression of pituitary appetite-regulating peptides including neuropeptide Y, cholecystokinin, melanin-concentrating hormone, ghrelin, and serotonin.(16) The pituitary has been reported to be responsible for modulating food intake.(17) Ghrelin, first isolated from the stomach, is a 28-amino acid, acylated, orexigenic peptide which stimulates the release of growth hormone from the pitupituitary, an effect that is distinct from the regulation by growth hormone-releasing hormone. (18) Sun et al(16) found that melaninconcentrating hormone and ghrelin mRNA levels were increased by Zn overdose, whereas food intake and neuropeptide Y mRNA levels were not affected. In this study, although the positive correlation between ghrelin and Zn concentration was not significant, serum ghrelin concentrations were lower in the children with low hair Zn concentrations. The lack of a significant correlation may be due to the fact that the number of subjects included in the study was small. Further studies investigating the relationships among Zn, ghrelin, melanin-concentrating hormone, neuropeptide Y and cholecystokinin are needed to explore these correlations.

In this study we measured concentrations of Zn in hair. Estimation of Zn in any single compartment for reliable assessment of Zn remains to be a major dilemma, as there is no single specific, sensitive, noninvasive assay of Zn in tissue or body fluid that can confidently and comprehensively assess the Zn status of the body. Serum Zn level, which indicates the Zn status of the moment, changes with the food spacing and many clinical disorders are known to be accompanied by decreased plasma or serum Zn content.(19) Hair Zn however is stable, does not fluctuate easily and the length of hair sampled can reflect a storage status of 3-4 months.(20) Its popularity lies in the fact that it can be collected easily and nontraumatically, can be stored easily and most trace elements have higher concentration in hair than other body compartment, which helps in the analytic process. Low hair Zn in children is always an indicator of low Zn status, while normal or high hair Zn levels may or may not be associated with the Zn status. (20, 21) Low Zn status becomes more obvious and reliable when a low hair Zn level is associated with low serum Zn in the same individual, indicating a chronic ongoing low Zn status.(21)

IGF-I actions are primarily modulated through binding to IGF binding proteins such as IGFBP-3. After synthesis in the liver, IGF-I and IGFBP-3 are secreted and remain tightly bound together in the circulation.(22) Preliminary evidence indicates that ghrelin exerts a marked stimulatory effect on plasma GH levels in both rats and humans.(23) In addition, it has been shown that the plasma levels of the acylated form of ghrelin may influence the age-related alterations in GH/IGF-I regulation.(24) Usually, decreased IGF-I and IGFBP-3 levels are accompanied by an increase in ghrelin levels. However our results do not support these negative correlations. This may be due to the fact that the study cohort was small. Additionally, our results may have been affected by the nature of the cases included in the study.

Serum ghrelin, IGF-I and IGFBP-3 levels should be monitored after re-establishment of normal Zn levels in support of the existence of a direct link among hair Zn concentrations, ghrelin, IGF-I and IGFBP-3 levels. Unfortunately there are no available data to this effect. Some animal studies have shown that ghrelin is not inversely related to circulating GH levels.(25, 26) Our results need to be confirmed by studis on larger groups and longitudinal investigations.

Although severe Zn deficiency in rats reduces circulating IGF-I and IGFBP-3 levels simultaneously,(27) Hall et al(22) showed that a graded model of dietary Zn deficiency had no effect on the levels of IGF-I, and IGFBP-3. Turgut et al(28) found that the addition of Zn to the diet of healthy rats had no effect on the levels of GH, IGFI, and IGFBP-3. However, studies in children clearly show that Zn increases IGF-I and IGFBP-3 levels.(29, 30) In a previous study, we found that Zn deficiency had a negative effect on growth hormone action on epiphyseal growth plates in rats.(31) We found that serum IGF-I and IGFBP-3 concentrations were positively related to the hair Zn concentrations. Since the relationship between Zn deficiency and reduced ghrelin concentration has not been described well in the literature, we speculated that poor appetite detected in the children with low Zn hair concentrations might be related to the reduced ghrelin concentrations.

In conclusion, we found that serum IGFI, IGFBP-3 and ghrelin concentrations were decreased in children with low hair Zn concentrations, and that serum IGF-I and IGFBP-3 concentrations were significantly correlated with hair Zn concentrations. Serum ghrelin ievels might also have been affected by low Zn concentrations in hair. Larger studies are needed to explore these relationships.
